# Association between Serum Leptin Concentrations and Insulin Resistance: A Population-Based Study from China

**DOI:** 10.1371/journal.pone.0054615

**Published:** 2013-01-22

**Authors:** Hui Zuo, Zumin Shi, Baojun Yuan, Yue Dai, Gaolin Wu, Akhtar Hussain

**Affiliations:** 1 Department of Nutrition and Food Hygiene, Jiangsu Provincial Center for Disease Control and Prevention, Nanjing, China; 2 Department of Community Medicine, Institute of Health and Society, Faculty of Medicine, University of Oslo, Oslo, Norway; 3 Discipline of Medicine, University of Adelaide, Adelaide, Australia; University of Colorado Denver, United States of America

## Abstract

**Background:**

Insulin resistance contributes to the cardio-metabolic risk. The effect of leptin in obese and overweight population on insulin resistance was seldom reported.

**Methods:**

A total of 1234 subjects (572 men and 662 women) aged ≥18 y was sampled by the procedure. Adiposity measures included BMI, waist circumference, hip circumference, WHR, upper arm circumference, triceps skinfold and body fat percentage. Serum leptin concentrations were measured by an ELISA method. The homeostasis model (HOMA-IR) was applied to estimate insulin resistance.

**Results:**

In men, BMI was the variable which was most strongly correlated with leptin, whereas triceps skinfold was most sensitive for women. More importantly, serum leptin levels among insulin resistant subjects were almost double compared to the subjects who had normal insulin sensitivity at the same level of adiposity in both men and women, after controlling for potential confounders. In addition, HOMA-IR increased significantly across leptin quintiles after adjustment for age, BMI, total energy intake, physical activity and smoking status in both men and women (p for trend <0.0001).

**Conclusions:**

There was a significant association between HOMA-IR and serum leptin concentrations in Chinese men and women, independently of adiposity levels. This may suggest that serum leptin concentration is an important predictor of insulin resistance and other metabolic risks irrespective of obesity levels. Furthermore, leptin levels may be used to identify the cardio-metabolic risk in obese and overweight population.

## Introduction

Obesity is now a global epidemic [1], [2]. It is one of the most significant causes of ill-health worldwide [3]–[5]. Anti-adiposity efforts to reverse the increasing trend are somewhat disappointing. However, all overweight or obese people do not have the same risk of developing insulin resistance, resulting in adiposity-related health risks such as type 2 diabetes, metabolic syndrome and cardiovascular diseases.

Leptin, an adipose tissue-derived hormone, activates researchers’ interest with a new insight since its discovery [6], [7]. It plays an important role in the pathophysiology of obesity [5]. Leptin acts centrally to decrease food intake and modulate glucose and fat metabolism [8].

Circulating leptin level was found to be proportional to adipose tissue mass, and body fat percentage was possibly the best adiposity-related predictor of serum leptin concentrations in human, which may be due to leptin resistance [9], [10]. However, it is not always easily accessible to directly measure the percentage of body fat, especially in epidemiological studies [11], [12]. In the studies on the association between leptin and indirect measures of adiposity, the most frequently used measures were body mass index (BMI) and waist circumference [13]–[16]. Very few studies [17], [18] reported information about surrogate dimension of other variables such as waist-to-hip ratio (WHR), arm circumference or triceps skinfold in relation to leptin. Also, it is unknown the proxy performance of body fat percentage estimated using prediction equation.

Although most surveys indicated a positive relationship of leptin and insulin resistance in their populations [16], [19], [20], others showed inconsistent results [14], [21], [22]. However, data on the effect of leptin in obese and overweight population on insulin resistance are scarce, other than one study focusing in diabetic women [14]. Furthermore, it remains unclear whether ethnic difference affects such association in addition to lifestyle factors such as smoking [13], [23].

Therefore, we have investigated the role of leptin in insulin resistance at different levels of obesity controlling for potential confounding factors including lifestyle and diet in a Chinese population.

## Methods and Subjects

### Study Design

Data used in this study were derived from the 2006 wave of the China Health and Nutrition Survey (CHNS) in Jiangsu Province. The CHNS study is a nationwide ongoing open cohort in China, started from 1989. More detailed information was described elsewhere [24]. Jiangsu was the only province that collected blood samples in that project in the 2006 wave. Therefore the study consisted of face-to-face questionnaire interviews, physical examinations, and laboratory analysis in Jiangsu. The study sample was drawn from six areas (two cities: Suzhou and Yangzhou; four counties: Shuyang, Taixing, Haimen, and Jinhu) following a multistage random cluster process. Socioeconomic status in these areas was a primary consideration. Further, 4 villages and townships in each county and 4 urban and suburban neighborhoods in each city were selected randomly. In total, 16 villages and townships within the counties and 8 urban and suburban neighborhoods within the cities were selected, respectively.

### Subjects

The study sample consisted of 572 men and 662 women aged ≥18 y from those household sampled by the procedure. Excluded were persons who had an age <18 y (n = 81), who had previously diagnosed diabetes (n = 48), who had any of missing anthropometric or leptin data (n = 59). The study was approved by the Review Board of Jiangsu Provincial Center for Disease Control and Prevention. All participants provided written consent. The subjects were compensated for their participation. The response rate was 91.3%.

### Adiposity Measures

Anthropometric data were measured by trained health workers following standard protocols. Weight in light clothing and without shoes was measured to the nearest 0.1 kg and height was measured to the nearest 0.1 cm. BMI was calculated as weight (kg)/height squared (m^2^). It was categorized as normal weight (BMI<24), overweight (BMI≥24 to <28), obese (BMI≥28) according to the Chinese standard [25]. Waist circumference was measured to the nearest 0.1 cm with an inelastic tape at the mid-way between the lowest rib and the iliac crest with the subject standing at the end of gentle expiration. Central obesity was defined as WC≥90 cm in men and ≥80 cm in women according to the International Diabetes Federation criteria [26]. Hip circumference was measured with the same tape to the nearest 0.1 cm at the maximum circumference over the buttocks. Upper arm circumference was measured to the nearest 0.1 cm with the left arm hanging relaxed. The measurement was taken midway between the tip of the acromion and olecranon process. Triceps skinfold was measured to the nearest mm in triplicate with a Lange skinfold caliper (Beta Technology Ltd, Cambridge, Maryland) having a pressure of 10 g/mm^2^ of contact surface area. The measurement was taken over the triceps muscle at the midpoint of the left posterior upper arm. Body fat percentage (BF %) was estimated using ethnic specific prediction equations: BF%  = 1.04*BMI – 10.9*sex +0.1*age +5.7 [where BMI = body mass index (kg/m2); sex: females  = 0, males  = 1; and age in years] for Chinese [27].

### Biochemical Analysis

Blood was collected by venipuncture from participants after overnight fasting. The fasting status was verbally confirmed by subjects before the blood sampling. All blood samples were collected in three vacuum tubes and processed within three hours. All specimens were then shipped to the Jiangsu Provincial Center for Disease Control and Prevention and were stored at −70°C for later laboratory testing. Fasting glucose was assessed by an enzymology method using OLYMPUS Chemistry Analyzer AU400 (Mishima Olympus CO., LTD, Shizuoka-ken, Japan). Insulin was measured by ELISA Kit (Millipore Corporation, Billerica, MA, USA). The homeostasis model assessment for insulin resistance (HOMA-IR) score was calculated as fasting insulin (mU/L) * fasting glucose (mmol/L)/22.5 [28]. Its highest quartile was used to define insulin resistance. Serum leptin concentrations were measured using Linco Human Leptin ELISA Kit (Linco Research, St. Charles, MO, USA), the sensitivity of which was 0.5 ng/ml – 100 ng/ml. The average intra- and inter-assay coefficients of variation were 4.7% and 7.2%, respectively.

### Diet, Physical Activity and Smoking Status Assessment

A semi-quantitative food frequency questionnaire (FFQ) [29] was used to collect dietary intake information. The questionnaire has been validated and compared with weighted food records and reported to be a useful method for the collection of individual food consumption information in face-to-face interviews, especially in studying the relationship between diet, nutrition and chronic diseases [29]. Participants were asked to recall their usual frequency and quantity of intakes of 33 food groups and beverages during the previous year with a series of detailed questions. Strict quality control procedures were took to minimize potential recall bias during the survey, which included well training of the survey workers, the use of food models, and so on. Intake of each food item was calculated by multiplying the reported frequency of the food by estimated portion size of the food per time. It was then converted into g/day for further analysis. Total energy intake was computed by using the Chinese Food Composition Table [30]. Physical activities including domestic, occupational, transportation and leisure-time physical activity were assessed in terms of metabolic equivalent (MET)-hours-per-week to account for both intensity and time spent on activities. The level of physical activity was the product of time spent in each activity multiplied by specific MET values based on the “Compendium of Physical Activities” [31]. Domestic physical activity was defined as activities such as food shopping for the family, food preparation and cooking, washing and ironing clothes and house cleaning. Occupational physical activity was defined as activities such as light-intensity (e.g., office work, counter salesperson, lab technician, etc.), moderate-intensity (e.g., driver, electrician) and vigorous-intensity physical activity (e.g., farmer, athlete, dancer, steel worker, construction worker). Transportation physical activity was defined by various transportation methods such as on foot, by bicycle, by bus/subway or by car/taxi/motorcycle. Leisure-time physical activity included martial arts, gymnastics/dancing/acrobatics, track and field (running, etc.)/swimming, soccer/basketball/tennis, badminton/volleyball and other (ping pong, Tai Chi, etc.). Current smoking status was classified as dichotomous variables (yes/no).

### Statistical Analysis

Distribution of leptin and HOMA-IR were highly skewed to the right, so geometric means and natural logs are presented in the analysis. Continuous variables were presented as means ± SEM. Means of serum leptin concentrations and HOMA-IR were generated and compared between the groups using the analysis of covariance (ANCOVA) procedure, adjusting for potential confounders. Pearson correlation coefficients were used to examine the association between serum leptin concentrations and different adiposity measures in men and women. The linear trend of adjusted geometric means of HOMA-IR across leptin quintiles was tested to assess the association between HOMA-IR and serum leptin concentrations in men and women after adjustment for age, BMI, total energy intake, physical activity and smoking status. Statistical analyses were performed separately for men and women, due to significant sex difference in serum leptin concentrations.

In addition, we generated predictive models for serum leptin concentrations and HOMA-IR, respectively, by using multivariate regression analysis. The candidate independent variables were gender, age, BMI, waist circumference, WHR, hip circumference, upper arm circumference, triceps skinfold, total energy intake, physical activity, smoking, insulin and HOMA-IR for serum leptin concentrations. The candidate independent variables were the same as above except substituting insulin and HOMA-IR to leptin for the prediction of HOMA-IR. Multivariate analyses excluded persons with missing values for any factor included in the model. Diagnostic measures including multicollinearity and interaction were examined in the predictive model. Interaction was performed by introducing production term into the model.

Stepwise procedure and adjusted R^2^ selection method were used for choosing optimal predictors. We also report the model R^2^ as a measure of the proportion of the variance in the values of dependent variable explained by the multivariate regression model. Only interaction terms were eligible in the model if it was significant, and had good predictive power (a larger model R^2^) and no multicollinearity. The values of p<0.05 were considered statistically significant. All analyses were conducted using the SAS (version 8.1, SAS Institute, Cary, NC).

## Results

Among 1234 study participants in our study, 416 (33.7%) were overweight and 118 (9.6%) were obese according to the Chinese criteria. Descriptive statistics of demographic, anthropometric and biochemical parameters for men and women are shown in Table 1. Women were a bit younger than men in general. There was no difference of BMI, hip circumference, physical activity and fasting glucose level between men and women. Men had higher waist circumference, WHR, upper arm circumference and a higher intake of total energy than women (p<0.0001). In contrast, triceps skinfold, body fat percentage, fasting insulin, HOMA-IR and leptin levels were significantly higher in women (p<0.0001). Smokers accounted for 51.9% in men and only 2.3% in women.

**Table 1:** Descriptive statistics of demographic, anthropometric and biochemical parameters in 1234 participants, stratified by sex.

	Men	Women	P value
n	572	662	
Age (years)	50.4±0.6	48.6±0.6	0.031
**Anthropometric measures** a			
* BMI (kg/m^2^)*	23.4±0.2	23.5±0.1	0.607
* Waist circumference (cm)*	83.6±0.4	79.2±0.4	<0.0001
* Hip circumference (cm)*	94.5±0.4	93.9±0.4	0.228
* WHR*	0.88±0.003	0.84±0.003	<0.0001
* Upper arm circumference (cm)*	27.5±0.1	26.5±0.1	<0.0001
* Triceps skinfold (mm)*	17.3±0.3	23.9±0.3	<0.0001
* BF (%)*	24.1±0.2	35.1±0.1	<0.0001
Total energy intake (kcal/d)^a^	2661±45	2242±42	<0.0001
Physical activity (MET-h/week)^a^	168.3±4.9	159.6±4.4	0.191
Smoking (%)	51.9	2.3	<0.0001
**Biochemical parameters** a			
*Fasting glucose (mmol/L)*	5.20±0.05	5.20±0.05	0.936
*Fasting insulin (uU/ml)*	5.47±0.21	6.58±0.19	<0.0001
*HOMA-IR* b	0.92±0.03	1.20±0.03	<0.0001
*Leptin (ng/ml)* b	1.45±0.05	8.32±0.05	<0.0001

BMI, body mass index; WHR, waist-to-hip ratio; BF (%), body fat percentage; HOMA-IR, homeostasis model assessment of insulin resistance.

Data are means ± SEM, unless otherwise indicated.

a Adjusted for age.

b Geometric means ± SEM.

Table 2 reports the correlation coefficients for men and women between serum leptin concentrations with different adiposity measures. Prior to adjustments, all adiposity parameters were significantly correlated with serum leptin concentrations (p<0.0001). In men, BMI had the strongest correlation with leptin, followed by waist circumference and BF (%). In women, the counterpart was triceps skinfold, followed by BMI and upper arm circumference. After controlling for age and BMI, all the variables except WHR in women remained significant. After further adjustment for age, total energy intake, physical activity, smoking status and HOMA-IR, the strong positive correlations still kept significant for all the variables in both men and women. In addition, serum leptin concentrations were more correlated with adiposity measures in women aged ≥52 y compared to those aged <52 y by stratified analysis (data not shown).

**Table 2:** Correlation analysis of serum leptin concentrations with different adiposity measures in men and women.

	Men (n = 572)			Women (n = 662)		
	Unadjusted	Model 1	Model 2	Unadjusted	Model 1	Model 2
BMI (kg/m2)	0.665***	–	0.521***	0.489***	–	0.372***
Waist circumference (cm)	0.637***	0.287***	0.483***	0.385***	0.119**	0.307***
Hip circumference (cm)	0.550***	0.198***	0.379***	0.352***	0.088*	0.280***
WHR	0.342***	0.129**	0.240***	0.196***	0.061	0.151**
Upper arm circumference (cm)	0.549***	0.196***	0.382***	0.422***	0.163***	0.399***
Triceps skinfold (mm)	0.481***	0.289**	0.392***	0.525***	0.350***	0.449***
BF (%)	0.615***	–	0.521***	0.410***	–	0.372***

Model 1, adjusted for age and BMI.

Model 2, adjusted for age, total energy intake (quintile), physical activity (quintile), smoking status (yes/no) and HOMA-IR (quintile).

Analysis was performed on log-transformed leptin concentration due to non-normality distribution.

* p<0.05,

** p<0.01,

*** p<0.0001.

**Figure 1:**
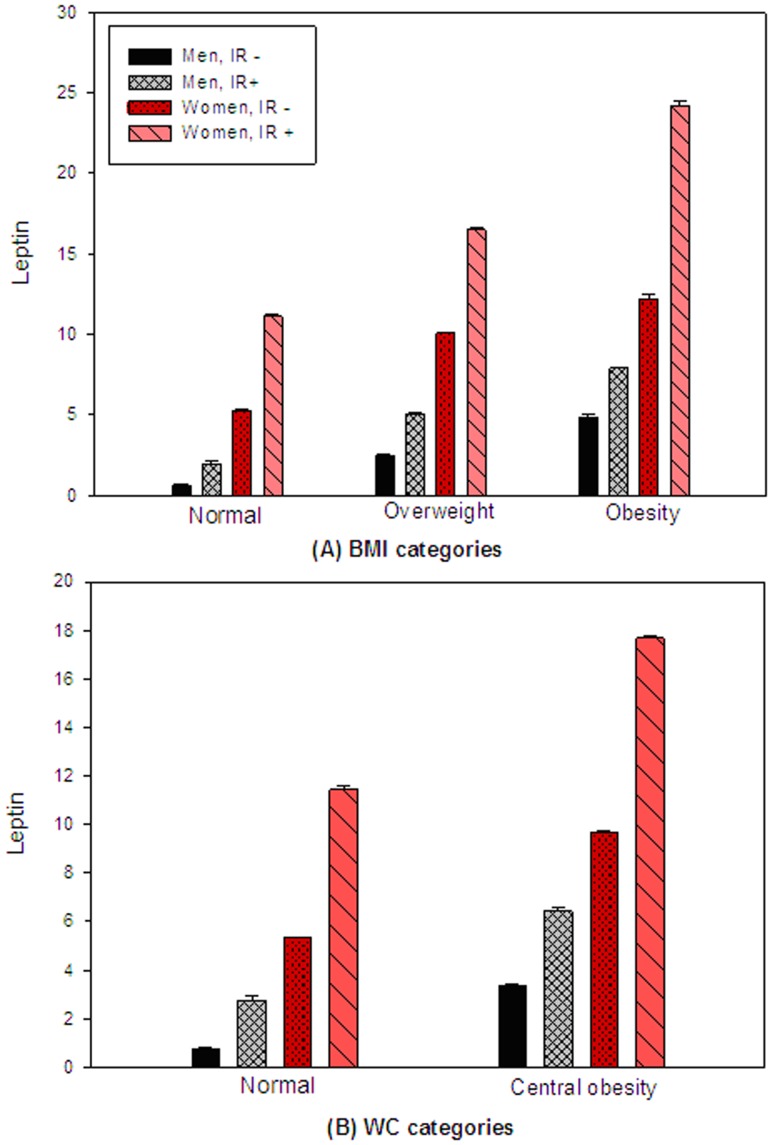
Serum leptin levels between participants with and without insulin resistance across different categories of adiposity in men and women, after adjustment for age, total energy intake (quintile), physical activity (quintile) and smoking status (yes/no). (Data are geometric means and SEM; Insulin resistance was defined as HOMA-IR >1.67 in men and >1.88 in women; P<0.001 between IR - and IR+groups at the same categories in men and women except P = 0.052 between two groups in obese women).

Figure 1 presents serum leptin levels between participants with and without insulin resistance across different categories of adiposity in men and women. After controlling for potential confounders, we found that participants with insulin resistance had significantly higher leptin levels compared to those without the condition, at all levels of adiposity, measured by BMI or waist circumference. The leptin level was almost double among the insulin resistance group compared to those without the condition.

**Figure 2:**
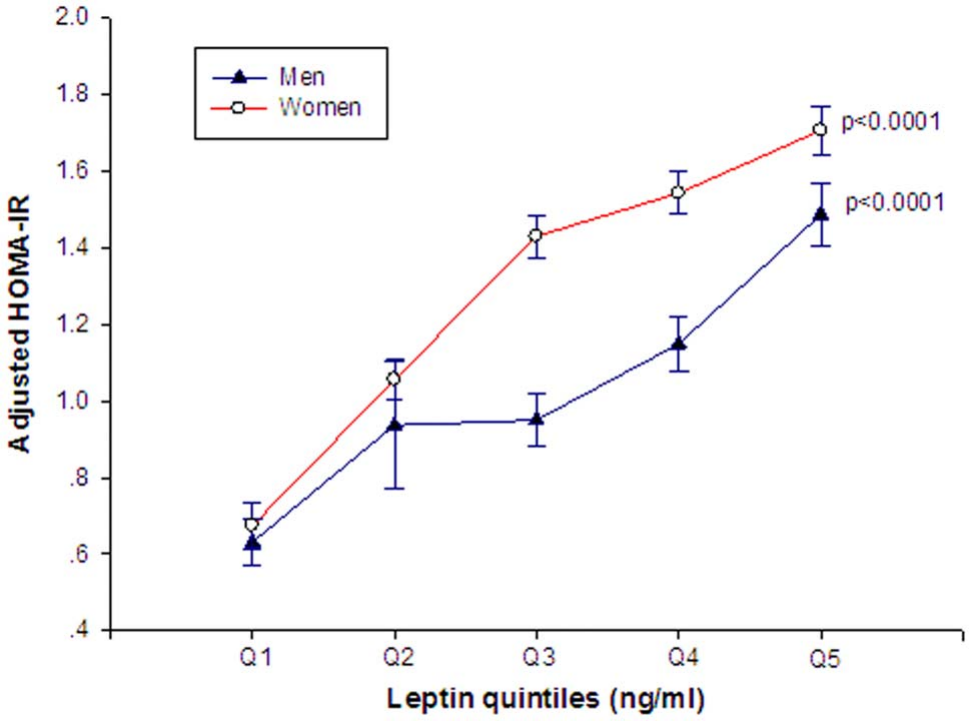
The association between HOMA-IR and serum leptin concentrations in men and women after adjustment for age, BMI, total energy intake (quintile), physical activity (quintile) and smoking status (yes/no). Geometric means ± SEM.

As shown in Figure 2, HOMA-IR increased significantly across leptin quintiles after adjustment for age, BMI, total energy intake, physical activity and smoking status in both men and women (p for trend<0.0001).

The serum leptin concentration was predicted by gender, age, BMI, waist circumference, triceps skinfold, total energy intake, physical activity, smoking, HOMA-IR and interaction term between total energy intake and smoking. Gender and HOMA-IR and triceps skinfold were the major determinants. All these independent variables explained 69% of the variance of leptin concentrations in the regression model. Moreover, in order to avoid potential over-adjustment, BMI, waist circumference and triceps skinfold were separately analyzed in model 1. We observed that all the ß coefficients of them were larger than 0.3 (Table 3).

**Table 3:** Multivariate regression analysis standardized ß coefficients (SE) for leptin concentration in Chinese adults in the 2006 CHNS study, Jiangsu.

	Model 1^a^			Model 2^b^		
	ß	SE	P value	ß	SE	P value
Gender	–	–	–	−0.404	0.073	<0.0001
Age (years)	–	–	–	−0.079	0.002	<0.0001
BMI (kg/m^2^)	0.330	0.009	<0.0001	0.135	0.012	<0.0001
Waist circumference(cm)	0.314	0.003	<0.0001	0.161	0.004	<0.0001
Triceps skinfold (mm)	0.317	0.004	<0.0001	0.218	0.004	<0.0001
Total energy intake(kcal/d, quintile)	–	–	–	−0.081	0.023	0.0001
Physical activity(MET-h/week, quintile)	–	–	–	−0.076	0.020	<0.0001
Smoking (yes/no)	–	–	–	−0.185	0.130	<0.0001
HOMA-IR	–	–	–	0.274	0.040	<0.0001
Energy*smoking	–	–	–	0.138	0.044	0.0003

Analysis was performed on log-transformed leptin concentration and HOMA-IR due to non-normality distribution.

a Model 1, BMI, waist circumference and triceps skinfold were put into the model separately, only information on the three adiposity measures was shown.

b Model 2, all independent variables in the table were put into the model simultaneously; Gender (0 women, 1 men); Smoking (0 no, 1 yes); Energy*smoking, interaction term between total energy intake (kcal/d, quintile) and smoking (yes/no); Model R^2^ = 0.69.

## Discussion

Our study demonstrated that leptin was independently associated with all measures of adiposity. Furthermore, our data suggest that among the overweight/obese population those with insulin resistance had a significant higher level of leptin concentration compared to those overweight/obese who were not insulin resistant even after controlling for a number of potential confounding factors. There was a gender difference in the association of various levels of adiposity measures with leptin. In men, BMI had the strongest association with leptin. While in women, it was triceps skinfold.

Diverse adiposity measures available in our study allowed us to compare the strength of the association with leptin. Despite of disparities existed, all adiposity measures were significantly correlated with serum leptin concentrations in both men and women. Overall, we found BMI, universally used as proxy indicator of general obesity, had a satisfactory performance to correlate with leptin, especially in men. Also, we found triceps skinfold was most strongly correlated with leptin in women. Our results are in agreement with two studies, one from US [17] and one from China [18]. Gender and age differences in the estimation of the strength of association with leptin may be explained by the effect of sex hormones [4]. In addition, the proxy performance of body fat percentage (BF %) calculated using prediction equation [27] was reported for the first time by our study.

Although a large number of studies have reported the association between HOMA-IR and serum leptin concentrations. Their interrelationship independent of adiposity level was not largely addressed. Most studies found a significant and independent association between HOMA-IR and leptin in their populations [16], [19], [20], which was confirmed by the present study. However, some studies reported inconsistent results [14], [21], [32].

Of note, we found that serum leptin levels among insulin resistant subjects were almost double compared to those subjects who were not insulin resistant at the same level of adiposity in both men and women. Our results are consistent with a study specifically focusing on diabetic women [14]. This finding may provide an insight into the explanation why the metabolic risk was different among persons with same degree of adiposity.

Regression analyses showed that lifestyles such as dietary energy intake, physical activity and smoking were all inversely associated with leptin, which is in line with previous studies [13], [23], [33]. Possible explanation for smoking is that nicotine might indirectly reduce leptin secretion via enhanced plasma catecholamine concentration [23]. The inverse association between physical activity and leptin concentrations may be explained by several mechanisms: leptin is proposed to activate the melanocortin-4 receptor in the arcuate nucleus and then influence physical activity; physical activity may also regulate the leptin level directly through reducing its synthesis or by improved insulin sensitivity [33].

In addition, results of prediction model building further confirmed that HOMA-IR and leptin were important determinants and predictors of each other. Letpin may play an independent role in developing insulin resistance and other metabolic risks. However, the casual relationship in this study cannot be identified owing to its design. Another limitation of the study is possible residual confounding due to indirect methods to estimate adiposity. However, the striking rise of overweight and obesity epidemic in the world requires special attention. Understanding the pathophysiological role of leptin and insulin resistance may help address the problem.

In summary, the association between leptin and insulin resistance was demonstrated irrespective of obesity levels. Further, significant higher level of leptin was found in insulin resistant subjects compared to the subjects without the condition at the same level of obesity in both gender. This finding may provide an insight into the explanation why the metabolic risk was different among persons with same degree of adiposity and may help identify the people at risk for diabetes and/or cardiovascular diseases across adiposity level and thereby an important contribution in clinical and preventive measures.
